# Diagnostic Difficulties in a Case of Persistent Cloaca with Hydrocolpos

**Published:** 2012-10-01

**Authors:** Zeki Sahinoglu, Aysenur Cerrah Celayir, Mehmet Resit Asoglu, Nahit Özcan

**Affiliations:** 1 Department of Obstetrics and Gynecology, Zeynep Kamil Women and Child Diseases Education and Research Hospital Istanbul, Turkey.; 2 Department of Pediatric Surgery, Zeynep Kamil Women and Child Diseases Education and Research Hospital Istanbul, Turkey.; 3 Sonomed Medical Imaging Center, Istanbul, Turkey.

**Keywords:** Persistent cloaca, Prenatal diagnosis, Fetal hydrometrocolpos, Fetal pelvic mass

## Abstract

Pelvic midline cystic mass associated with renal malformation represents typical imaging features of a cloacal anomaly. We report a case of persistent cloaca that was diagnosed antenatally with fetal ultrasonography and MRI.

## INTRODUCTION

Cloacal malformations can be diagnosed reliably in the prenatal period. Persistent cloaca with an incidence of 1 in 50.000 births can be recognized due to the presence of pelvic midline cystic structures [1, 2]. When hydronephrosis, hydrometrocolpos, intrapelvic fluid collections, dilated distal bowel, spinal and/or sacral deformities, or absent kidney are noted in a female fetus, it is highly suggestive of a cloacal malformation. Technical advances related to the in-utero diagnosis are expected to improve the accuracy of the prenatal diagnosis [3]. Prenatal detailed evaluation is essential for counseling the family and planning follow-up [3, 4]. 

We report a case of persistent cloaca diagnosed by ultrasound and fetal MRI and highlight the role of MRI in providing some additional valuable clinical information.

## CASE REPORT

A 40-year-old pregnant woman, gravida 5, para 2, abortus 2 was referred to our prenatal diagnosis center due to oligohydramnios. Her obstetric history was unremarkable. A single fetus at the 34 weeks of gestation was detected by ultrasonography. On the detailed fetal scan, fetal bladder could not be visualized, but a small thick walled pelvic cystic mass of 17x16 mm size was seen just in the region of bladder (Fig. 1A). Two tubular, cystic, separate structures of sizes 17x30 and 16x32 mm were detected just behind the cyst described above. Fetal genitalia were indistinguishable. 

**Figure:**
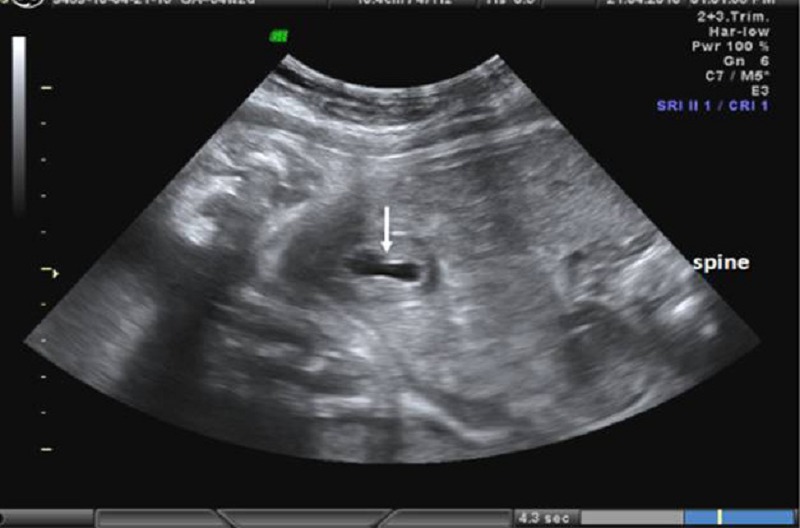
Figure 1A: A cystic thick walled pelvic mass (white arrow) was detected in the supra-pubic region instead of the bladder.

**Figure:**
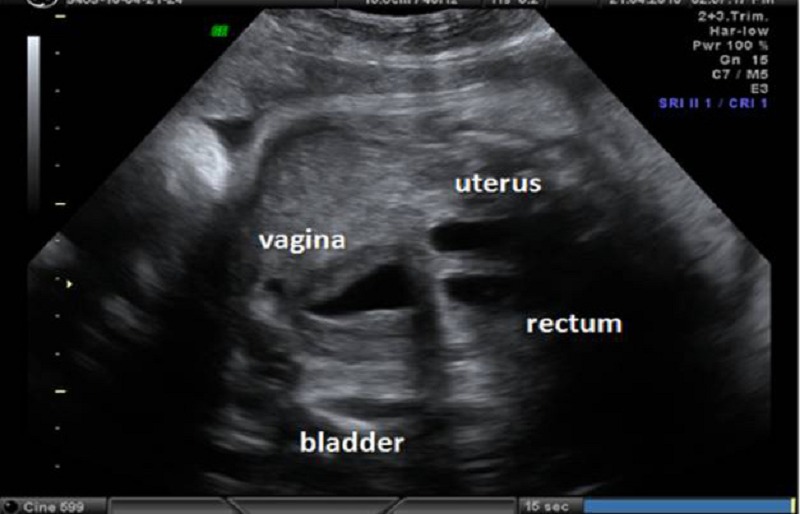
Figure 1B: Two fusiform cystic masses were appeared during the scan just behind the pubic cystic mass described in Figure 1 A. The walls of these 2 structures were less thick than the former.

**Figure:**
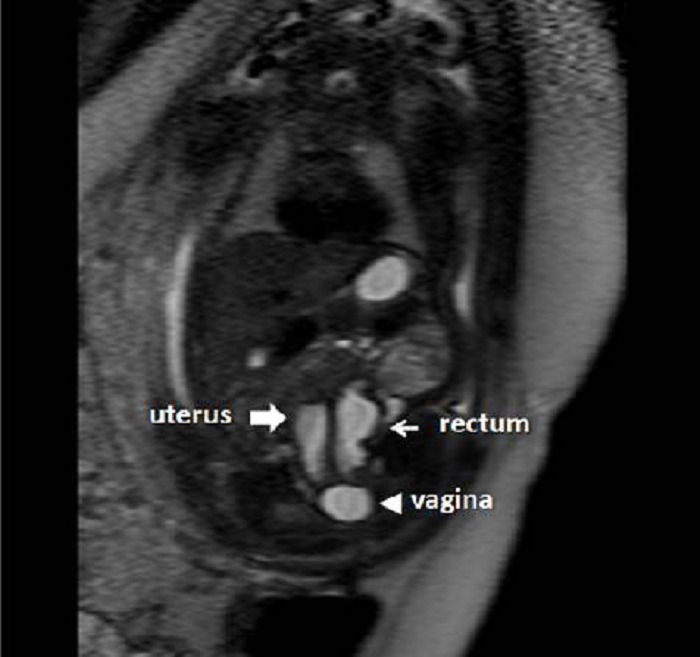
Figure 2A: A dilated vagina, uterus and rectum were demonstrated by fetal MRI. A separate bladder was invisible.

A prenatal diagnosis of persistent cloaca was made; the cystic structures were diagnosed as dilated vagina, hydrometrocolpos and dilated rectum respectively. The compressed, blurred bladder was noticed inferior to the dilated vagina (Fig. 1B). 

Additional findings included unilateral left-sided hydroureteronephrosis and the right renal agenesis. The amniotic fluid index was calculated as 72 mm. Fetal magnetic resonance imaging (MRI) confirmed all the sonographical findings except the right sided renal agenesis; the right kidney was found severely hypoplastic and distorted (Fig. 2A, 2B). The family was counseled regarding the fetal malformations. Due to fetal distress, a caesarian section was performed at the 36 weeks of gestation and a baby weighing 2700g was delivered.

**Figure:**
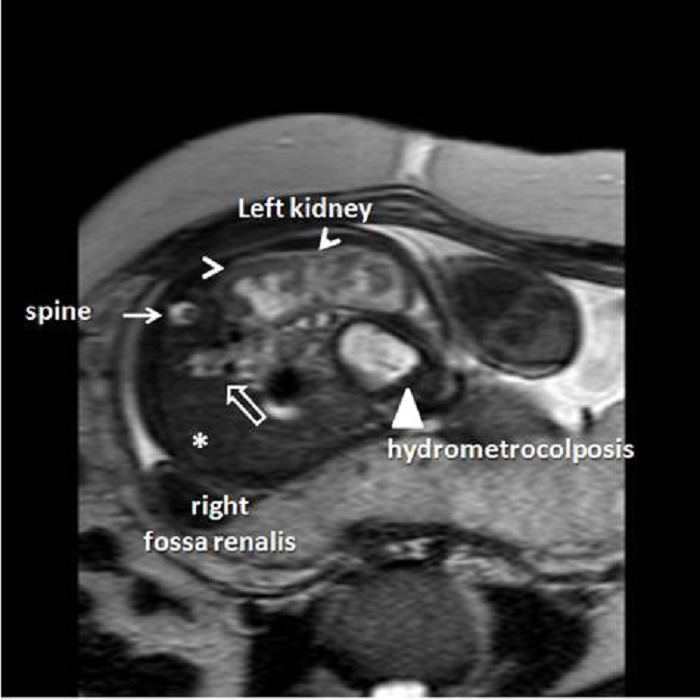
Figure 2B: Left hydronephrotic kidney (white arrow heads) was demonstrated in fetal MRI while right fossa renalis (white asterix) was empty. However, just behind the spine, a structure with a similar appearance to a dysplastic, slightly distorted right kidney was reported (white empty arrow). Hydrometrocolposis was clearly visualized by fetal MRI (white triangle).

At birth, a single orifice was present in the perineum suggestive of persistent cloaca. At the postnatal ultrasonographic examination, hydrometrocolposis, dilated rectum, left hydroureteronephrosis and right renal agenesis were confirmed. Colostomy and abdominal vaginostomy were done. Her kidney functions stabilised over next two months. She is waiting for a definitive operation.

## DISCUSSION

Accurate diagnosis and classification of cloacal anomalies during pregnancy is essential to predict the perinatal morbidity and mortality [5, 6, 10]. The differential diagnosis of pelvic mass in a female fetus includes hydrocolpos, meconium pseudocyst, enteric duplication cyst, fetal ovarian cyst, dilated bowel, bowel duplication, mesenteric cyst, rectal duplication, cystic neuroblastoma, or bladder duplication. The diagnosis of hydrometrocolpos should hint on the presence of anomalies such as imperforate hymen, urogenital sinus, persistent cloaca [3, 5-10]. 

Common channel in cloaca is sometimes atretic resulting in rectal, uterine and/or vesical distension. This anomaly may be difficult to assess with fetal US. Hydronephrosis may occur and the bladder may be poorly visualized. The mixing of urine and meconium may cause apparition of calcifications in the intestinal lumen, which are visible with US [1, 9]. MRI, on the other hand, provides better anatomic resolution, regardless of fetal position. However, there are no large studies published comparing the efficiency of fetal ultrasound and MRI in persistent cloaca [1, 4, 6, 7-9]. In a study comparing the diagnostic advantages of prenatal ultrasound and MRI in pelvic mass, it was reported that the cephalad extension detected in fetal MRI was misdiagnosed by ultrasound in some fetuses [1, 6-9]. However, the authors mentioned that trans-abdominal Doppler sonography was sufficient for assessing the extension of cystic and extra=pelvic mass [1, 6-9]. The appearance of small bladder with unilobulated or bilobulated pelvic cystic masses in association with the enlarged rectum and mild to moderate oligohydramnios in prenatal ultrasonography is strongly suggestive for persistent cloaca [1, 9]. Its location and internal heterogeneous part corresponding to a tissue component were reported as characteristic MRI features consistent with internal pelvic cystic organs. Our initial diagnosis of cloacal malformation was confirmed by MRI. MRI could pick up hydrometrocolposis, large rectum and small bladder, so as we could make a more accurate prenatal diagnosis of hydrometrocolpos due to persistent cloaca. The postnatal features were consistent with fetal MRI findings. We found fetal MRI superior to trans-abdominal ultrasound in this case. 

In our case, the bladder was visualized with the umbilical arteries around and two cystic structures were detected behind it. These intrapelvic cystic structures were thought to be hydrocolpos and dilated rectum due to the direct communication between the urinary tract and the genital and gastrointestinal tracts via the common canal of persistent cloaca. In the following scans, a small thickened urinary bladder was seen along with a distended vagina. Although it has been reported that a hydrocolpos can be differentiated from the bladder by the presence of a fluid-debris level formed by the presence of urine mixed with uterovaginal secretions and/or meconium, the contents of both cystic structures behind the bladder were in hypoechogenic, fluid filled and had clear appearances in our case.

Accurate prenatal diagnosis has a crucial role in the perinatal management and planning surgical options in pelvic cystic masses. The fetal imaging techniques could facilitate counseling the parents.

## Footnotes

**Source of Support:** Nil

**Conflict of Interest:** None
